# Key gut bacterial taxa and their correlative relationships with host genes during *Musca domestica* aggregation

**DOI:** 10.3389/fmicb.2026.1760699

**Published:** 2026-07-01

**Authors:** Ting Li, Zhang Kexin, Jinxiao Li, Qian Zhang, Yansong Yin, Ruiling Zhang, Zhong Zhang

**Affiliations:** 1 School of Life Science, Shandong First Medical University (Shandong Academy of Medical Sciences), Tai’an, Shandong, China; 2 Hospital for Skin Diseases, Shandong First Medical University, Tai’an, China; 3 Shandong Provincial Institute of Dermatology and Venereology, Shandong Academy of Medical Sciences, Tai’an, China; 4 School of Basic Medical Science, Shandong First Medical University (Shandong Academy of Medical Sciences), Jinan, Shandong, China; 5 Shandong Second Medical University, Weifang, Shandong, China

**Keywords:** group rearing, gut microbiome, housefly, larval growth and development, transcriptome

## Abstract

Aggregation enhances survival and reproduction by regulating body coloration, physiology, and immunity. The housefly (*Musca domestica*) is a notable sanitary pest that exhibits group-breeding behavior during its larval stage, which promotes its growth and development. However, the mechanisms underlying how high larval density regulates growth remain unclear. Previous studies have shown that gut bacteria are important regulators of larval growth and development. To further investigate this, the present study aimed to assess the impact of group rearing on both the gut microbiota and host gene expression in housefly larvae. The results showed that the relative abundance of the gut bacterial genera *Enterococcus*, *Myroides*, and *Serratia* significantly decreased in aggregated larvae compared with single larvae, and the relative abundance decreased as population density increased. Transcriptome analysis revealed that differentially expressed genes were significantly enriched in the lysosome pathway, wherein most genes were aspartic protease genes. The correlation network between gut bacteria and genes demonstrated that aspartic protease genes were closely correlated with changes in the intestinal bacteria. This study identifies intestinal bacterial genera and host genes influenced by population density, lays the foundation for further research on the mechanism underlying that group rearing improves larval growth, and is also expected to offer novel perspectives for pest control through the regulation of population density.

## 1 Introduction

Animal aggregation is a widespread phenomenon in the wild that offers survival advantages to individual animals. Through group living, organisms benefit from enhanced growth, feeding efficiency, defense against adversaries, and increased opportunities for mating and reproduction (Elliot and Hart, 2010; Qian et al., 2024; Wu et al., 2024). Gregarious behavior is commonly observed in insects, such as the aggregation of locusts and German cockroaches (Guo et al., 2023; Wada-Katsumata et al., 2015). The housefly (*Musca domestica*), a sanitary pest worldwide, poses substantial public health risks by serving as a vector for various disease-causing pathogens. The larvae thrive in groups, arising from the aggregation of ovipositing adult flies and from their vulnerable nature and restricted living environments (Wu et al., 2024). Group living is fundamental and advantageous for the growth and development of the larvae (Cičková et al., 2013; Kökdener and Kiper, 2021).

The mechanisms underlying these density-dependent effects, however, remain unclear. One possible mediator is the gut microbiota, which is known to be sensitive to environmental and social factors. Previous studies have provided considerable evidence for the significant influence of gut microbiota, including both beneficial and pathogenic taxa, on the growth and development of housefly larvae (Chen et al., 2020). In these studies, bacterial taxa were isolated from the housefly gut, cultivated, and incorporated into the larval diet. The experimental results demonstrated that gut bacteria, such as *Escherichia coli*, *Klebsiella pneumoniae*, and *Proteus mirabilis*, positively influence larval growth and development (Zhang et al., 2023; Zhang et al., 2022; Zhang et al., 2021b). Conversely, detrimental taxa, such as *Pseudomonas aeruginosa* and *Serratia marcescens*, have been found to negatively affect larval growth and development by influencing humoral immune responses, phenol oxidase activity, and intestinal tissue integrity (Li H. et al., 2023; Zhang et al., 2021a). Thus, the premise that gut bacteria play a significant role in housefly larval growth and development is well supported by experimental evidence. These physiological changes are often underpinned by alterations in host gene expression, suggesting a potential link between microbiota-mediated effects and the larval transcriptome. Given that these bacterial effects on host physiology often involve the regulation of host genes, it is plausible that density-dependent changes in the gut microbiota could drive corresponding transcriptional responses in the larvae.

However, despite this evidence, it is still unclear whether gut bacteria mediate enhanced larval growth and development in response to high larval density; furthermore, their interaction with host genes remains underexplored. To address this gap in the literature, this study aimed to test the hypothesis that high larval density promotes growth and development by altering the gut bacterial community and host gene expression. Specifically, we sought to confirm that group rearing promotes the larval growth and development of houseflies and identify the vital gut bacterial genera and genes altered by high larval density. This work will not only advance our understanding of the impact of population density on gut bacteria but also contribute to pest management strategies.

## 2 Materials and methods

### 2.1 Animals

Houseflies were reared in gauze cages (30 cm × 30 cm × 30 cm) covered with breathable gauze at Shandong First Medical University. The culture environment was controlled by an artificial climate incubator, with the humidity maintained at 70 ± 5%, the temperature controlled at 25 ± 1°C, and the duration of light and dark was 12 h, respectively, as described in a previous study (Li et al., 2024). To ensure synchrony in larval development, eggs were collected within a strictly limited 2-h time window. The Ethics Review Committee of Shandong First Medical University approved this study protocol (No. W202311130309) and the animal welfare has been maximally protected in this project.

### 2.2 Single and group rearing

Hatched housefly larvae of the same age were divided into two groups. One group was the single rearing group, in which a single housefly larva was placed in a 5 mL culture tube with small hole covered by gauze on the cover, and fed a diet mass of 1 mg. The other group was the group rearing group, in which 30 housefly larvae were co-reared in a 50 mL culture tube with hole covered by gauze on the cover and a diet mass of 10 mg. The diet consisted of sterilized wheat bran, milk powder and sterilized water (10:1:10). The diet supplied for larvae in both groups was same, thoroughly mixed. The amount of food per larva was not matched between the two groups since this design was aim to keep similar air-to-food volume ratios and humidity, rather than to provide equal food per individual. As the results show (section 3.1), food was sufficient in both conditions: group-rearing larvae grew better and uneaten food remained in their tubes. Both groups were placed in the same cage under the same culture conditions. In the density experiment, four population densities of 10, 20, 30, and 50 individuals per tube were set and the rearing condition was as the same as the group rearing group. These densities were chosen to create a gradient around the standard group density (30 per tube), allowing observation of density-dependent trends without causing extreme overcrowding.

### 2.3 Measurement and sampling

Two batches of larvae under single and group rearing were set for 16S rRNA and transcriptome sequencing (Supplementary Figure 1). At the same time on each of the second, third, fourth, and fifth days of rearing, 15 larvae were measured from the single-rearing group (one larva per tube, from 15 tubes) and 15 larvae from the group-rearing group (five larvae per tube, from three tubes). For both rearing conditions, the 15 measured larvae were then divided into three biological replicates for 16S rRNA sequencing and transcriptome sequencing, each replicate consisting of five larvae. In the single-rearing condition, the five larvae in each replicate came from five different single-rearing tubes. In the group-rearing condition, the five larvae in each replicate came from the same group-rearing tube. Including 30 larvae (3 replicates) on the first day (newly hatched larvae), one batch of larvae was sampled for 16S rRNA sequencing, along with the feed samples. Similarly, the other batch of larvae on the first, second and third day was sampled for transcriptome sequencing. An additional batch of larvae was reared until eclosion to measure pupation and eclosion ratios.

### 2.4 16 S rRNA sequencing and data analysis

Housefly larvae were sampled after two rinses with sterile water and an intermediate rinse with 75% ethanol. Total DNA including intestinal microbiota DNA was extracted after the larvae were homogenized in a lysis buffer. The hypervariable V3–V4 region of the bacterial 16S rRNA gene was amplified and sequenced. Data analysis included sequence assembly, chimeric sequence detection, Operational Taxonomic Unit (OTU) clustering, and linear discriminant analysis effect size (LEfSe). All above were performed as described in a previous study (Li et al., 2021). For 16S rRNA sequencing, three biological replicates were analyzed for each rearing condition on each time point. For group-rearing group, each biological replicate consisted of five larvae pooled from the same rearing tube, with each rearing tube as one biological replicate. For single-rearing group, each biological replicate consisted of five larvae pooled from five separate rearing tubes (one larva per tube). The FASTQ files produced by sequencing were submitted to the National Center for Biotechnology (NCBI) Sequence Read Archive (SRA) under the BioProject accession numbers PRJNA 1136129 and PRJNA1136139.

### 2.5 Short time-series expression miner (STEM) analysis

STEM was used for expression pattern analysis of intestinal bacterial genera (Ernst and Bar-Joseph, 2006). The imported data consisted of the abundance data for all intestinal genera of housefly larvae at different densities in the density experiment. After logarithmic normalization, expression data were clustered using the STEM clustering method. The maximum number of model profiles was set to 27, and the maximum unit change in the model profiles between time points was set to three.

### 2.6 Transcriptome sequencing and data analysis

Total RNA was extracted using the TRIzol reagent and the cDNA library was constructed and sequenced by Novogene company, on an Illumina HiSeq 4000 platform with a 150-bp read length. Clean data were mapped to the genome of *M. domestica* (Diptera: *Muscidae*) using the read aligner HISAT2 (version 2.0.5) and assembled using StringTie (version 1.3.3) as described in previous studies (Li et al., 2024). For transcriptome sequencing, three biological replicates were analyzed for each rearing condition on each time point, following the same replicate definition as described for 16S rRNA sequencing. Simultaneously, transcripts were annotated and their expression levels were evaluated. Differentially expressed genes (DEGs) were identified using the edgeR (R-3.2.4) package in R when the false discovery rate (FDR) < 0.05 (Benjamini-Hochberg correction) was < 0.05 and the absolute fold change was > 2.0. FASTQ files of the transcriptome sequences for 15 samples of housefly larvae are available at the NCBI SRA under the BioProject accession number PRJNA1135921.

### 2.7 Kyoto encyclopedia of genes and genomes (KEGG) analysis

KEGG analysis was performed using the KOBAS 3.0 web server.^1^ The hypergeometric test/Fisher’s exact test was used for statistical analysis. False discovery rate (FDR) was controlled using the Benjamini–Hochberg procedure. Pathways with adjusted *p*-value (*q*-value) < 0.05 were regarded as significantly enriched.

### 2.8 Pearson correlation analysis and correlation network construction

Pearson correlation analysis was performed using the cor(), cor.test(), and p.adjust() functions in the R programming language. The percentage values of OTU for genus biomarkers and fragments per kilobase million (FPKM) of differentially expressed genes were used as input data. Relationships with absolute correlation value > 0.6 and adjusted *p*-value (*q*-value) < 0.05 were selected for constructing correlation networks using Cytoscape software (v3.7.1).

### 2.9 Real-time quantity PCR (qPCR) on intestinal bacteria and host genes

The qPCR was performed by using ChamQ SYBR qPCR Master Mix (Catalog No. Q311-02, Vazyme, Nanjing, China) on a Light Cycler 480 instrument (Roche). The relative abundance of intestinal bacteria or expression levels of the genes were calculated using the 2^–Δ^*^Ct^* method. Unique amplification of target bacterial genus or genes was confirmed by melting curves and sequencing of the qPCR products. Supplementary Table 1 lists the primers used.

For intestinal bacteria, DNA from larvae on the third day under single- or group-rearing condition was isolated by VAMNE Magnetic Stool/Soil DNA Extraction Kit (Catalog No. DMA5101, Vazyme, Nanjing, China). Then, qPCR validation on the abundance of intestinal bacteria was performed with the bacterial DNA as the template and eight independent biological replicates were used for each group. ΔCt = Ct (target bacterial genus in sample *i*)—Ct (total bacteria in sample *i*). The total abundance of intestinal bacteria was presented by V3–V4 region of the bacterial 16S rRNA gene.

For host genes, 1 mg of total extracted RNA, which was extracted using the TRIzol reagent, was reverse transcribed to cDNA with random primers by using Moloney murine leukemia virus reverse transcriptase (Catalog No. M1705, Promega, Madison, WI). Three independent biological replicates were used for each group. GAPDH was used as the endogenous reference gene. ΔCt = Ct (target gene in sample *i*)—Ct (GAPDH gene in sample *i*).

### 2.10 Statistical analysis

For statistical analyses of 16S rRNA and transcriptome sequencing data, each biological replicate was treated as an independent experimental unit. In the group-rearing condition, each biological replicate corresponded to a single rearing tube; in the single-rearing condition, each biological replicate pooled larvae from five separate rearing tubes. Student’s *t*-test was used to analyze differences in housefly larval body weight and length, alpha diversity of intestinal bacteria, the abundance of bacterial genus and expression of host genes in qPCR. The chi-square test was used to assess the pupation and eclosion rates.

## 3 Results

### 3.1 Group rearing promotes the larval growth and development of housefly

**FIGURE 1:**
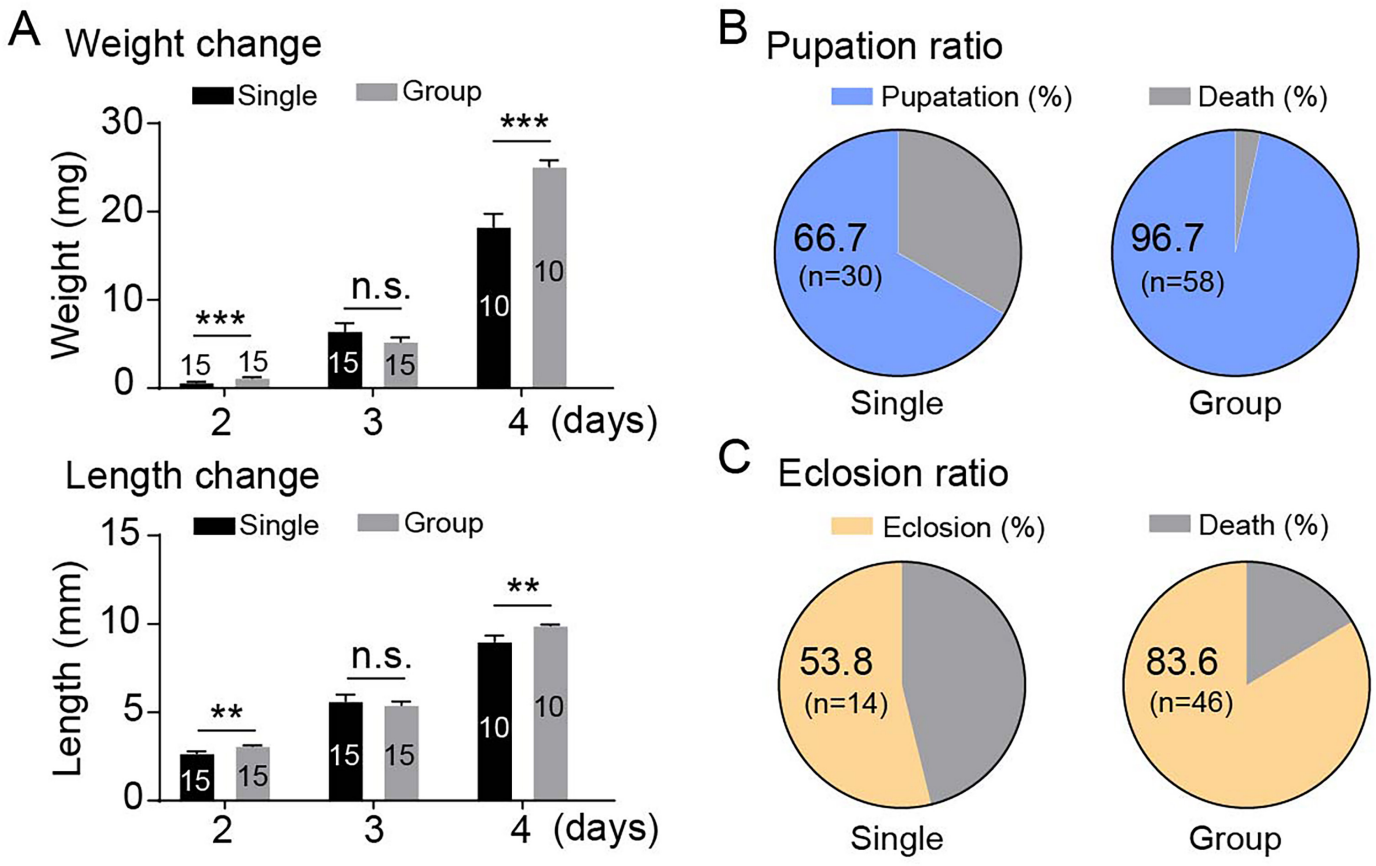
Larval growth and development of the housefly under single- and group-rearing conditions. **(A)** The changes in body weight (the upper) and length (the lower) of larvae when reared under single and group conditions during 4 days of rearing. **(B,C)** Pupation ratio **(B)** and eclosion ratio **(C)** of larvae under single- and group-rearing conditions. Measurements in **(A)** are shown as the mean ± SE. Statistical significance: n.s., not significant; **P < 0.01; ***P < 0.001.

On the 2nd and 4th days of rearing, the weight (Student’s *t*-test, *p* < 0.001 and *p* < 0.001) and body length (Student’s *t*-test, *p* = 0.0013 and *p* = 0.019) of larvae in the group-rearing condition were significantly higher than those in the single rearing condition (Figure 1A and Supplementary Tables 2, 3). Continuing to rear the larvae until pupation and eclosion revealed that the pupation ratio (Chi-square test, *x*^2^ = 10.3, *p* = 0.0013) and eclosion ratio (Chi-square, *x*^2^ = 5.0, *p* = 0.026) of larvae in the group-rearing condition were also significantly higher compared to those reared individually (Figures 1B,C). The above results demonstrate that group rearing can promote larval growth and development, and increase pupation and eclosion ratios.

### 3.2 Intestinal microbiota composition of single- and group-rearing larvae

The feed consumed by single-rearing larvae often grew mold in the later stages of rearing, whereas this was rare in group rearing. Therefore, we performed 16S rRNA sequencing to analyze the microbiota composition of the environment in which the larvae lived and fed, as well as the intestinal microbiota. Only on the 5th day but not the 2nd to 4th day, were richness and diversity of the environmental microbiota in group-rearing larvae significantly higher than those in single-rearing larvae, indicating a more complex and stable microbiota structure in gregarious larvae (Supplementary Figures 2A,B). In the environmental microbiota, the dominant bacterial genus during the first 3 days was *Pseudomonas*, whereas the abundance of *Providencia* and *Myroides* gradually increased over the last 2 days (Supplementary Figure 3).

**FIGURE 2:**
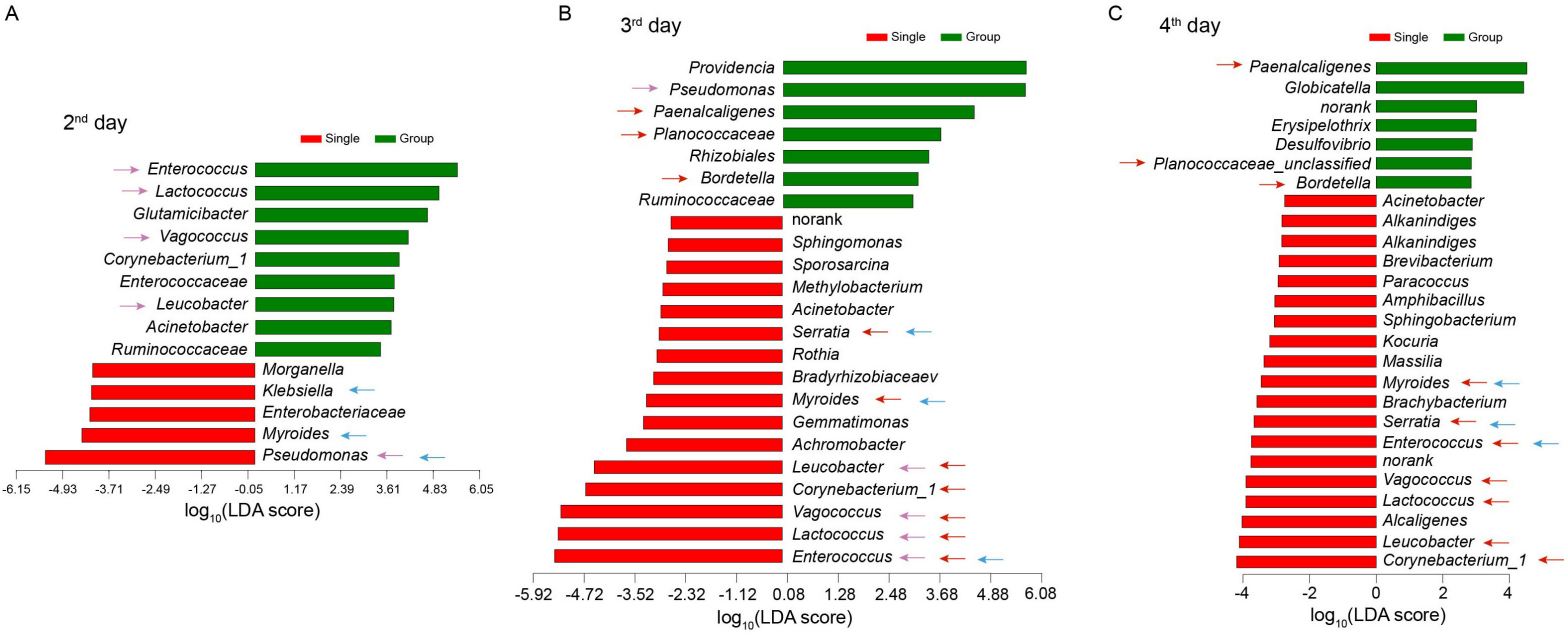
Differences in larval intestinal bacterial communities at the genus level between single and group rearing. **(A–C)** LEfSe analysis of larval intestinal genera under single and group rearing on the 2nd day **(A)**, the 3rd day **(B)**, and 4th day **(C)**. The criteria for biomarker identification were LDA > 2 and *p* < 0.05. Purple arrows represent intestinal bacterial genera showing opposite abundance patterns between single- and group-reared larvae on the 2nd and 3rd days. Red arrows represent intestinal bacterial genera that act as biomarkers on both the 3rd and 4th days. Blue arrows represent gut bacterial genera whose abundance varied significantly with density in the density experiment.

The richness and diversity of the bacterial genera in the environment were lower than those in the gut from the 2nd to 5th days (Supplementary Figure 2). Because intestinal rather than environmental bacteria are directly related to larval development, we focused on the gut microbiota. Although there was no significant difference in the richness and diversity of the intestinal bacterial taxa between single and group rearing (Supplementary Figures 2C,D), the abundances of bacterial genera were significantly different. LEfSe analysis revealed that nine bacterial genera were more abundant during group rearing, whereas five bacterial genera were more abundant during single rearing (LDA > 2, *p* < 0.05) on the 2nd day (Figure 2A). On the 3rd and 4th days, a total of 23 and 26 bacterial genera showed different abundances between single- and group-rearing larvae (LDA > 2, *p* < 0.05), respectively (Figures 2B,C). On both the 3rd and 4th days, 10 bacterial genera showed significant differences in abundance between single and group rearing (Figures 2B,C). Interestingly, the relative abundances of the four bacterial genera were reversed between single and group rearing from the 1st day to the 2nd day (Figures 2A,B).

These results indicate that the group rearing of housefly larvae significantly altered the gut microbiota, and the abundance of several gut bacterial genera exhibited marked differences between single and group-rearing. Significant differences in gut bacteria may be associated with the enhanced larval growth and development in response to group rearing.

### 3.3 Population density impacts the composition of the larval gut microbiota

Four population densities of 10, 20, 30, and 50 individuals per tube were used in the density experiment, and the composition of larval intestinal bacteria on the third day was analyzed by 16S rRNA sequencing. At all rearing densities, the dominant intestinal bacterial genus was *Providencia* (Supplementary Figure 4A). Notably, the gut microbiota composition of larvae reared at a density of 10 was distinctly different from that at other densities. Richness, an alpha diversity metric, did not exhibit significant differences across rearing densities (*p* = 0.24, 0.44, and 0.11; Supplementary Figures 4B,C). However, the gut microbiota diversity at densities of 20, 30, and 50 was significantly lower than that at a density of 10 (*p* = 0.006, 0.02, and 0.002, respectively; Supplementary Figures 4D,E). This suggests that increased population density may inhibit the survival and proliferation of certain bacterial genera, leading to a reduction in gut microbiota diversity.

**FIGURE 3:**
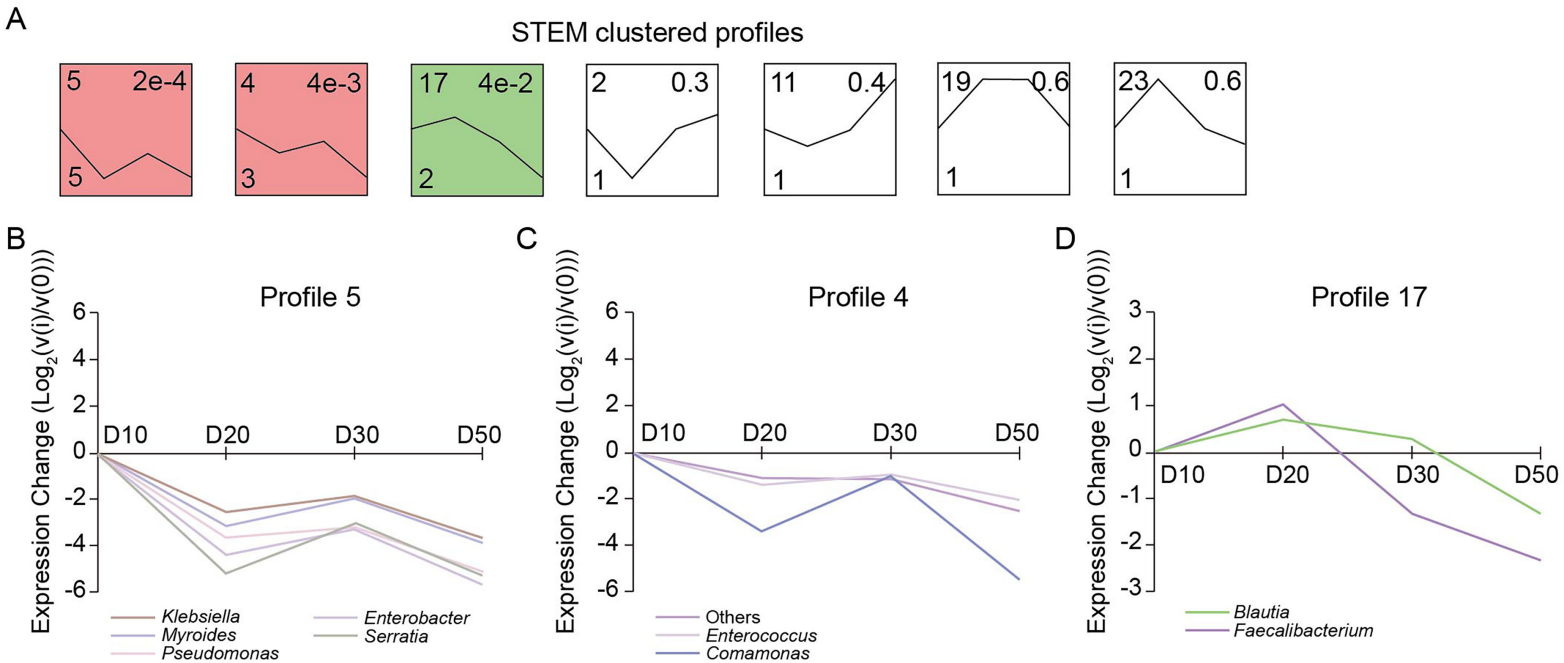
Short time-series expression miner (STEM) analysis of the changes in abundance of larval intestinal bacterial genera across different population densities. **(A)** The clustered profiles of change patterns of the larval intestinal bacterial genera using STEM. The colored profiles were significant (FDR < 0.05). The numbers in the top left corner, top right corner, and bottom left corner represent the module ID, significance values, and number of included genera, respectively. **(B–D)** The expression changes of bacterial genera in the significantly clustered profiles 5 **(B)**, 4 **(C)**, and 17 **(D)**. The v(0) and v(i) represent the abundance of bacteria genera at the population density of 10 and i individuals per tube, respectively.

Using the STEM program, we analyzed the changes in gut bacterial genus abundance as rearing density increased. Three significantly clustered expression profiles were identified: profiles 5 (*p* < 0.001), 4 (*p* = 0.004), and 17 (*p* = 0.04) (Figure 3A). The bacterial genera clustered within these profiles were significantly influenced by rearing density, and their abundance decreased as density increased. In profile 5, which was the most significantly enriched profile, five gut bacterial genera, *Klebsiella*, *Myroides*, *Pseudomonas*, *Enterobacter*, and *Serratia*, were identified (Figure 3B). Profiles 4 and 17 contained two clustered bacterial genera, respectively (Figures 3C,D).

Among these significantly clustered gut bacterial genera, five showed differences in abundance between single and group rearing (Figures 2A–C, 3B,C). Moreover, three genera, *Enterococcus*, *Myroides*, and *Serratia*, exhibited consistent abundance differences on both the 2nd and 3rd days (Figures 2B,C, 3B).

### 3.4 Differentially expressed genes were enriched in the lysosome pathway

**FIGURE 4:**
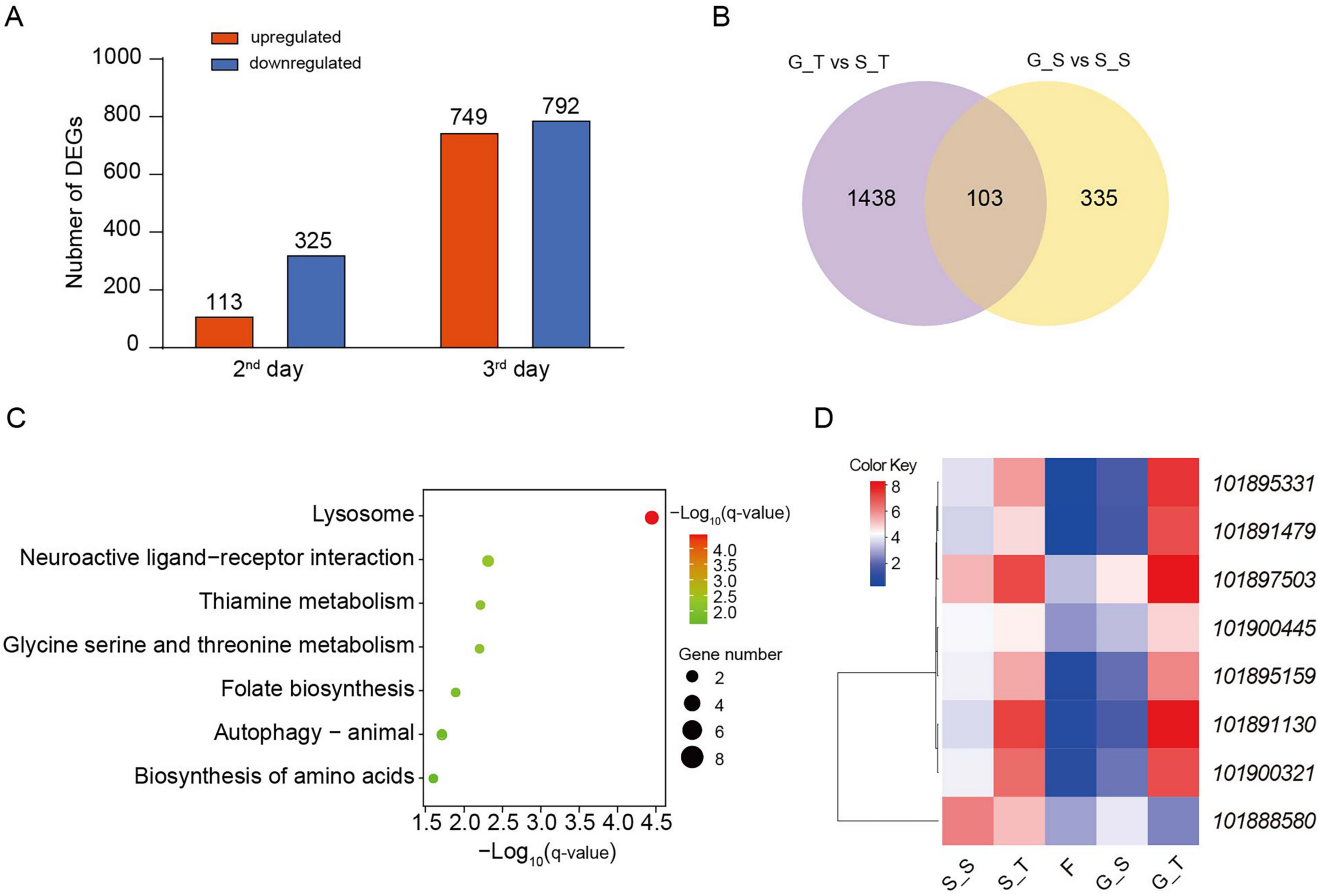
Transcriptome analysis of the housefly larvae under single and group rearing. **(A)** The number of up- and down-regulated genes among DEGs. **(B)** Venn diagram of DEGs. **(C)** Enriched pathways of overlapping DEGs on the 2nd and 3rd days using KEGG analysis. The pathways with larger and dark red circles contained more DEGs and were more significant. **(D)** Expression heatmap of genes enriched in the lysosome pathway. Darker red indicates a higher expression level.

The transcriptome of five groups, including one group of larvae on the first day (F); two groups of single-rearing larvae on the second (S_S) and third (S_T) day, respectively, and two groups of group-rearing (30 individuals per tube) larvae on the second (G_S) and third day (G_T), respectively, were sequenced. Sample clustering analysis demonstrated that three biological replicates within each group clustered closely together, indicating the high reliability of sampling (Supplementary Figure 5A). A total of 438 and 1,541 differentially expressed genes were identified between single and group rearing on the 2nd and 3rd days, respectively (Supplementary Figure 5B). The number of DEGs on the 2nd day was less than that on the 3rd day (Figure 4A). There were 103 overlapping DEGs on the 2nd and 3rd days (Figure 4B). These overlapping DEGs were significantly enriched in the lysosome pathway (*q* = 3.56E-5, Figure 4C). Eight DEGs were enriched in the lysosome pathway; their expression heatmap is shown in Figure 4D. Among these, *101895331*, *101891479*, *101895159*, and *101891130*, were annotated as aspartic protease genes.

### 3.5 Correlation analysis between the intestinal bacteria and the host genes

**FIGURE 5:**
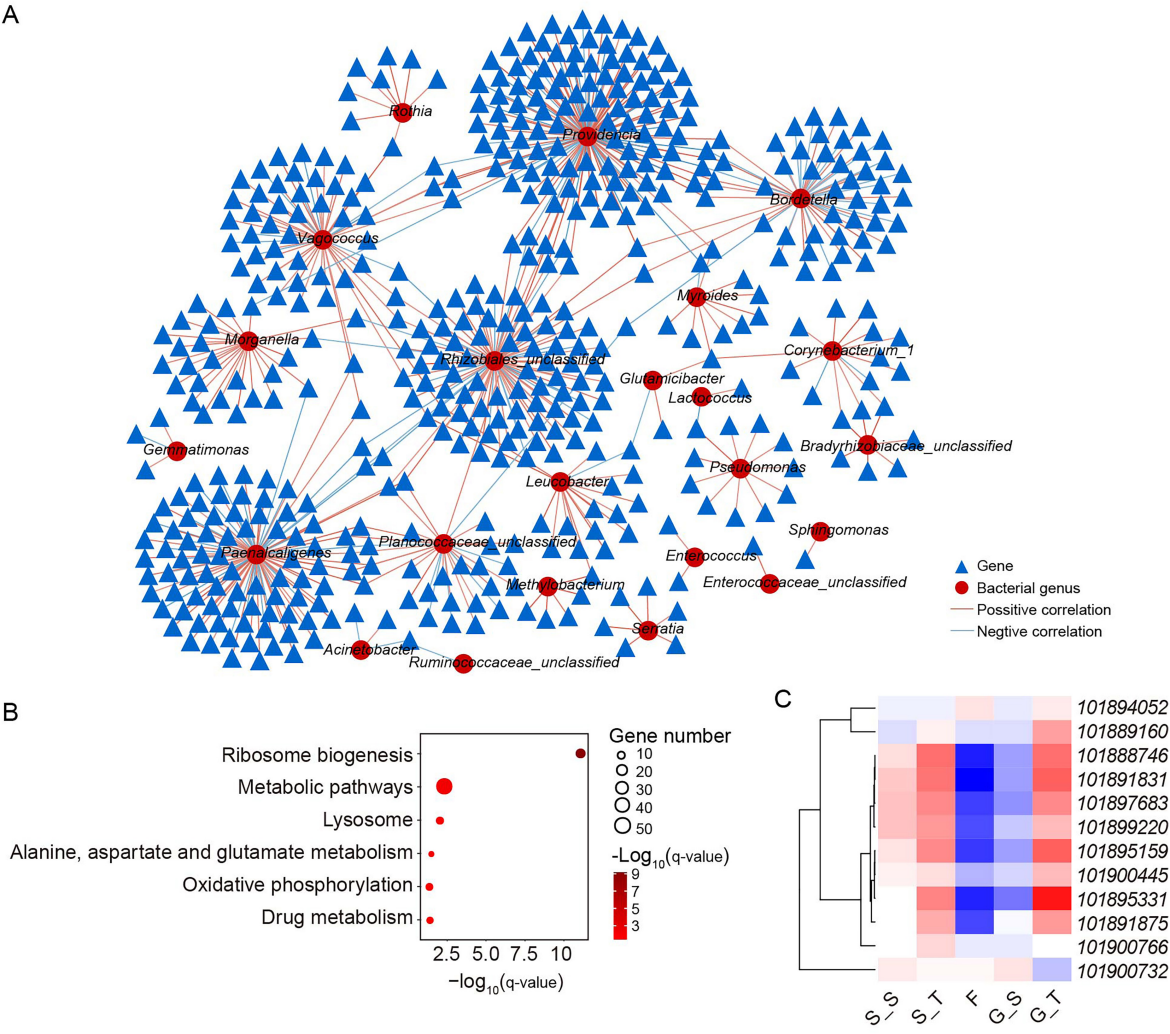
Co-expression network between intestinal bacterial genera and DEGs. **(A)** The network of correlation between intestinal bacterial genera biomarkers and DEGs under single and group rearing (density of 30). The Pearson correlations with |*r*| > 0.6 and *q*-value < 0.05 are shown in the network. Circles represent bacterial genera, while the triangles represent genes. The edges in red and blue indicate positive and negative correlations, respectively. **(B)** Enriched KEGG pathways of genes associated with bacterial genera biomarkers. **(C)** Expression heatmap of genes enriched in the lysosome pathway.

The co-expression correlation based on the abundance of gut bacterial genus and host gene expression from the 15 samples of single and group rearing (density of 30) was analyzed, and a network was constructed to uncover the relationship between intestinal bacteria and host genes. In the co-expression networks, 555 correlations (|*r*| > 0.6 and *q* < 0.05) were identified (Figure 5A). All related host genes were subjected to pathway enrichment analysis, which revealed that the lysosome pathway was significantly enriched again (*q* = 0.015, Figure 5B), suggesting a strong correlation between intestinal bacterial abundance and the expression of lysosome-related genes. In this pathway, twenty-two genes were enriched, and aspartic protease gene *101895331*, and *101895159* were also differentially expressed on both the 2nd day and the 3rd days (Figures 4D, 5C).

### 3.6 qPCR validation of intestinal bacterial abundance and host gene expression

**FIGURE 6:**
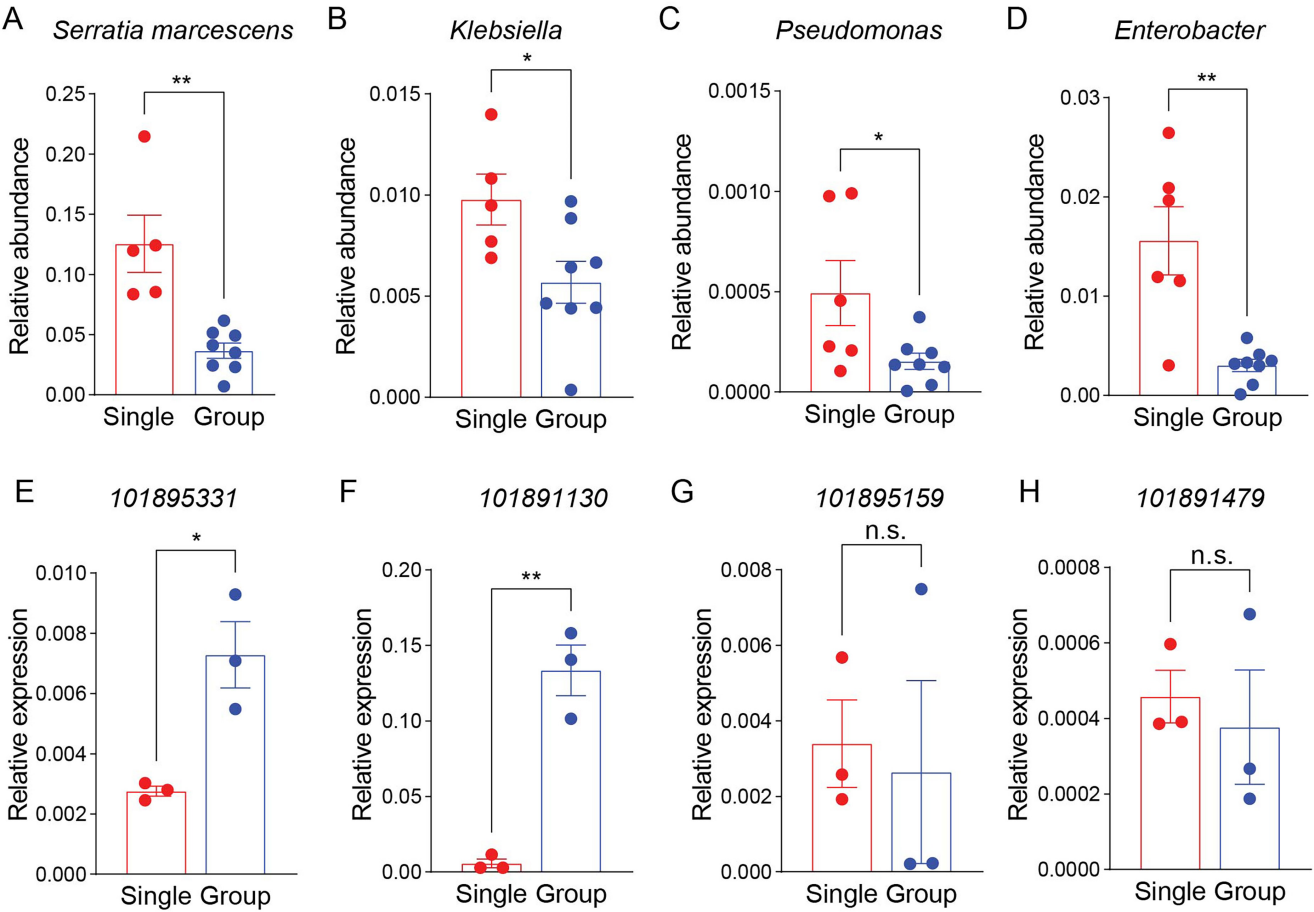
The abundance of intestinal bacteria and expression level of aspartic protease gene in single- and group-rearing group. **(A–D)** The relative abundance of *Serratia marcescens* **(A)**, *Klebsiella* **(B)**, *Pseudomonas* **(C)** and *Enterobacter* **(D)**. **(E–H)** The relative expression of *101895331* **(E)**, *101891130* **(F)**, *101895159* (**G**) and *101891479* **(H)**. Measurements are shown as the mean ± SE. Statistical significance: n.s., not significant; *P < 0.05; **P < 0.01.

To confirm the abundance of intestinal bacteria and expression level of aspartic protease gene, real-time qPCR was performed. The relative abundances of intestinal bacteria, which were clustered in Profile 5 of STEM analysis (Figure 3B), *Serratia marcescens*, *Klebsiella*, *Pseudomonas*, and *Enterobacter* were notably higher in the single-rearing group than group-rearing (Student’s *t*-test, *p* = 0.001, 0.03, 0.04, and 0.001, for *Serratia marcescens*, *Klebsiella*, *Pseudomonas*, and *Enterobacter*, respectively, Figures 6A–D). The relative expressions of aspartic protease gene which were enriched in the lysosome pathway (Figure 4D) were also validated. Among them, *101895331* and *101891130* were significantly higher in the group-rearing group (Student’s *t*-test, *p* = 0.02 for *101895331*, *p* = 0.002 for *101891130*, Figures 6E,F) while that of *101895159* and *101891479* were not significantly different between single-and group-rearing (Student’s *t*-test, *p* = 0.79 for *101895159*, *p* = 0.65 for *101891479*, Figures 6G,H).

## 4 Discussion

### 4.1 The group rearing of house fly larvae influenced the composition of environmental and intestinal microbiome

Gut microbiota have been shown to influence insect host in various aspects, such as olfaction, locomotion, learning, memory, and the production of chemical pheromones (Moyano et al., 2023). For instance, the gut microbiota of Colorado potato beetles altered their feeding behavior by modulating the expression of odor receptors (Li H. et al., 2023). Gut symbionts in German cockroaches contribute to aggregation by producing volatile carboxylic acids (Wada-Katsumata et al., 2015). In this study, density gradient experiments highlighted nine gut bacterial genera whose abundance was significantly correlated with larval density, five of which showed significant differences in abundance between single and group rearing. In particular, *Serratia*, *Myroides*, and *Enterococcus* exhibited sustained differences in abundance over consecutive days, suggesting that they are closely related to high larval density and may enhance larval growth under group rearing. This work implies that host behavior, such as aggregation, can also alter the composition of the gut microbiota and highlights population density as an important factor shaping the gut microbiota.

In the experiment, the diet was sterilized for both single- and group-rearing larvae which were reared from the same cohort of first-instar larvae, ensuring comparable initial environmental and gut microbial conditions. Notably, the richness of environmental microbiota under group-rearing was higher. This may be explained, firstly, by the fact that the initial environmental microbiome was contributed by multiple larvae rather than a single individual; although these larvae were from the same cohort, subtle individual variations may have introduced greater bacterial diversity into the substrate. Additionally, a subsequent group effect may further shape this community, wherein aggregated feeding, high moisture gradients, low oxygen availability, and secretions suppress certain dominant bacterial strains, thereby enhancing the survival opportunities of other bacterial species (Cifuentes et al., 2020; Henry et al., 2020). Interestingly, despite the higher environmental microbial diversity, group-rearing larvae exhibited lower gut microbial diversity. This contradiction may be also attributed to group-induced physiological stress or intraspecific competition, potentially altering larval digestive or immune processes and leading to selective inhibition of specific environmental bacteria within the intestinal tract (Shan et al., 2024; Upfold et al., 2023). The transition from a highly diverse environmental microbiota to a comparatively restricted gut microbiota is a complex and poorly understood phenomenon that warrants further exploration. Moreover, the influence of environmental microbiota on larval gut microbiota and physiology remains an important topic for future research.

One notable observation during the experiment was that mold frequently developed on the feed of singly-reared larvae but was rarely seen under group rearing. This difference is unlikely to be due to the amount of leftover food, as both conditions had excess feed. Instead, we attribute it to the gregarious activities of group-reared larvae, including continuous physical disruption of the food surface, metabolic heat accumulation, possible secretion of antimicrobial substances, and a more diverse environmental microbiota that may suppress fungal growth. This phenomenon further illustrates how group living alters the local micro-environment in ways that extend beyond the larval gut microbiota.

### 4.2 *Pseudomonas* and *Serratia* showed correlated abundance patterns with larval growth and development under group rearing

Previous studies have experimentally demonstrated that *Pseudomonas aeruginosa* and *Serratia marcescens* adversely affect housefly larval growth. Supplementation of larval diet with these bacteria reduces growth rate, whereas phage-mediated reduction of *S. marcescens* promotes growth (Li Y. et al., 2023; Zhang et al., 2021a). Importantly, *S. marcescens* has been shown to interfere with gut microbial composition, inhibit phenol oxidase activity, and cause intestinal damage (Li Y. et al., 2023). In this study, group-reared larvae, which exhibited enhanced growth, had lower abundances of *Pseudomonas* and *Serratia* compared to single-rearing larvae. This inverse pattern is consistent with the known growth-suppressive effects of these bacteria. These prior findings make it biologically plausible that the density-dependent reduction of *Serratia* and *Pseudomonas* observed in our study contributes to the enhanced growth of group-rearing larvae. Therefore, although our data are correlative, the consistency with prior functional studies lends support to this hypothesis. Future validation could be achieved using bacterial supplementation and phage-mediated targeted depletion (as in Li Y. et al., 2023), rather than germ-free models, as complete sterility may not be appropriate for testing detrimental bacteria.

Alternative explanations for the observed density-dependent effects were also considered. Host-driven processes cannot be excluded: larval density might directly modulate growth via endocrine or behavioral pathways, independent of gut microbiota. However, the strong associations between gut bacterial composition and density suggest that microbiota may participate in or reflect these effects. Regarding developmental asynchrony, larval hatching was strictly synchronized using a 2-h egg collection window, and larvae were sampled at the same time each day, ensuring comparable developmental stages between single- and group-rearing groups. Environmental influences were minimized by maintaining identical temperature and humidity, and by using sterilized food and the same initial environmental microbiota for both conditions; the differences that emerged later are precisely the consequence of density-dependent processes. These alternative explanations do not negate the strong correlations observed in this study, but rather highlight important directions for future research.

### 4.3 Aspartic protease genes were associated with larval intestinal microbiome under group rearing

Transcriptomic analysis revealed that the lysosome pathway is closely related to the larval group rearing, particularly the aspartic protease genes in this pathway. Lysosomes are cellular organelles that degrade biomolecules such as proteins, nucleic acids, and polysaccharides using various hydrolases, including proteases (Perera and Zoncu, 2016; Yang and Wang, 2021). Aspartic proteases, which function in lysosomes, cytosol or as secretory enzymes, play a significant role in protein degradation and proenzyme activation (Benes et al., 2008; Di et al., 2021). For example, aspartic proteases (Cathepsin D) of the triatomine *Rhodnius prolixus*, are important regulators of yolk protein degradation during the embryogenesis (Gomes et al., 2010). Study on silkworm *Bombyx mori* found that aspartic proteases acted as a metamorphosis-specific lysosomal proteinase to regulate histolysis of larval fat body and larval gut (Gui et al., 2006). During *Helicoverpa armigera* metamorphosis, aspartic protease was demonstrated to promote midgut apoptosis by cleaving and activating caspase 3 protein inside midgut cells (Di et al., 2021). Considering the above studies on insect aspartic protease and the enrichment in the lysosome pathway, the aspartic protease genes are possibly involved in intracellular digestion or immune processes (e.g., antimicrobial peptide activation) to indirectly regulate the gut bacteria of house fly. On the other hand, in the Muscidae family, specifically in houseflies, secretory aspartic proteases (e.g., Gene *101895159* found in this study) have been reported to be secreted into the midgut, where they act as protein hydrolases and are thought to be involved in food digestion (Padilha et al., 2009; Sojka et al., 2008; Terra et al., 2019). However, whether they directly interact with or affect gut bacteria was unknown.

Although the correlation network analyses identified aspartic protease genes as candidate regulators on gut bacterial composition, it should be noted that the correlation network analysis presented here is exploratory and does not establish causal relationships. Functional validation (e.g., gene knockdown/overexpression) is needed to determine their relationships. Whether the aspartic proteases identified in this study act intracellularly or extracellularly, and how they influence the gut environment and physiology of the larvae, remain open questions.

## 5 Conclusion

This study identified gut bacterial genera, particularly *Serratia*, *Myroides*, and *Enterococcus*, and lysosome pathway genes that are associated with group rearing, and revealed the correlations between gut bacteria and host genes under high larval density. It is important to note that the present findings are primarily correlative and not causal. Any mechanistic interpretations are presented as hypotheses that require future experimental validation. Future research should include manipulation of candidate core microbiota, and functional validation of lysosome pathway genes. These approaches will be crucial to unravel the mechanistic pathways through which group rearing influence larval growth via the gut microbiome, and contribute to the development of eco-friendly pest management strategies.

## Data Availability

The datasets presented in this study can be found in online repositories. The names of the repository/repositories and accession number(s) can be found at: https://www.ncbi.nlm.nih.gov/, PRJNA 1136129; https://www.ncbi.nlm.nih.gov/, PRJNA1136139; https://www.ncbi.nlm.nih.gov/, PRJNA1135921.

## Footnotes

1
http://bioinfo.org/kobas
