# Supplementary material for: Key gut bacterial taxa and their correlative relationships with host genes during Musca domestica aggregation
Source: Front Microbiol. 2026 Jul 1;17:1760699. doi: 10.3389/fmicb.2026.1760699 (PMC13370678; doi:10.3389/fmicb.2026.1760699)
Supplement: Supplementary file 1 [file Data_Sheet_1.DOCX]

**Supplementary information for “Key gut bacterial taxa and their correlative relationships with host genes during *Musca domestica* aggregation”**

**Ting Li^1*†^, Kexin Zhang^2, 3†^, Jinxiao Li^4†^, Qian Zhang^4^, Yansong Yin^4^, Ruiling Zhang^4*^, Zhong Zhang^1, 4, 5*^**

^1^ School of Life Science, Shandong First Medical University (Shandong Academy of Medical Sciences), Tai’an 271016, Shandong, China

^2^ Hospital for Skin Diseases, Shandong First Medical University

^3^ Shandong Provincial Institute of Dermatology and Venereology, Shandong Academy of Medical Sciences

^4^ School of Basic Medical Science, Shandong First Medical University (Shandong Academy of Medical Sciences), Jinan, Shandong, China

^5^ Shandong Second Medical University, Weifang 261021, Shandong, China

*** Correspondence：**

Zhong Zhang, E-mail: nasonia@163.com

Ruiling Zhang, E-mail: rlzhang@tsmc.edu.cn

Ting Li, E-mail: litingsdfmu@163.com

**^†^**These authors have contributed equally to this work.

**Supplementary Figures:**

**
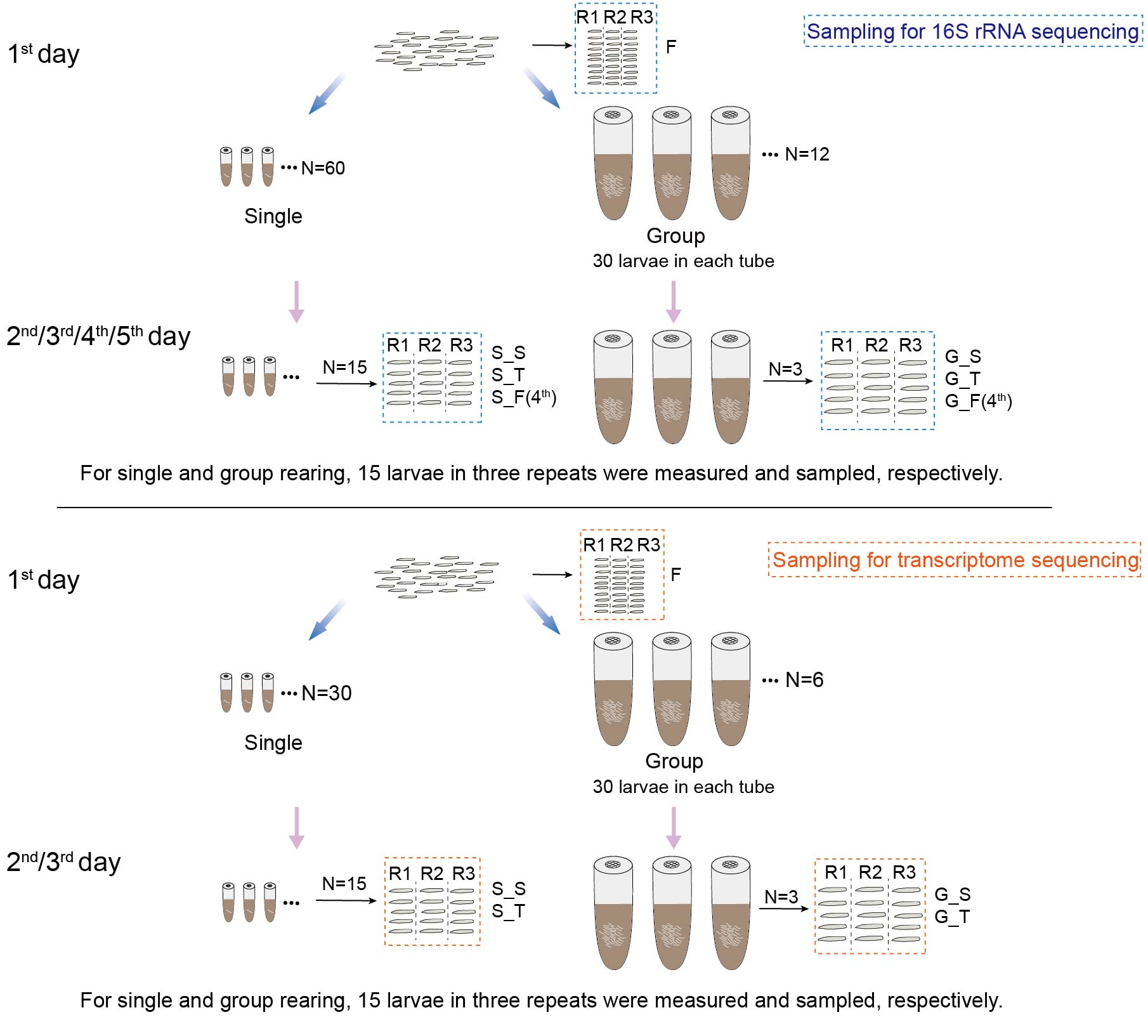
**

**Figure S1 Rearing and sampling scheme**


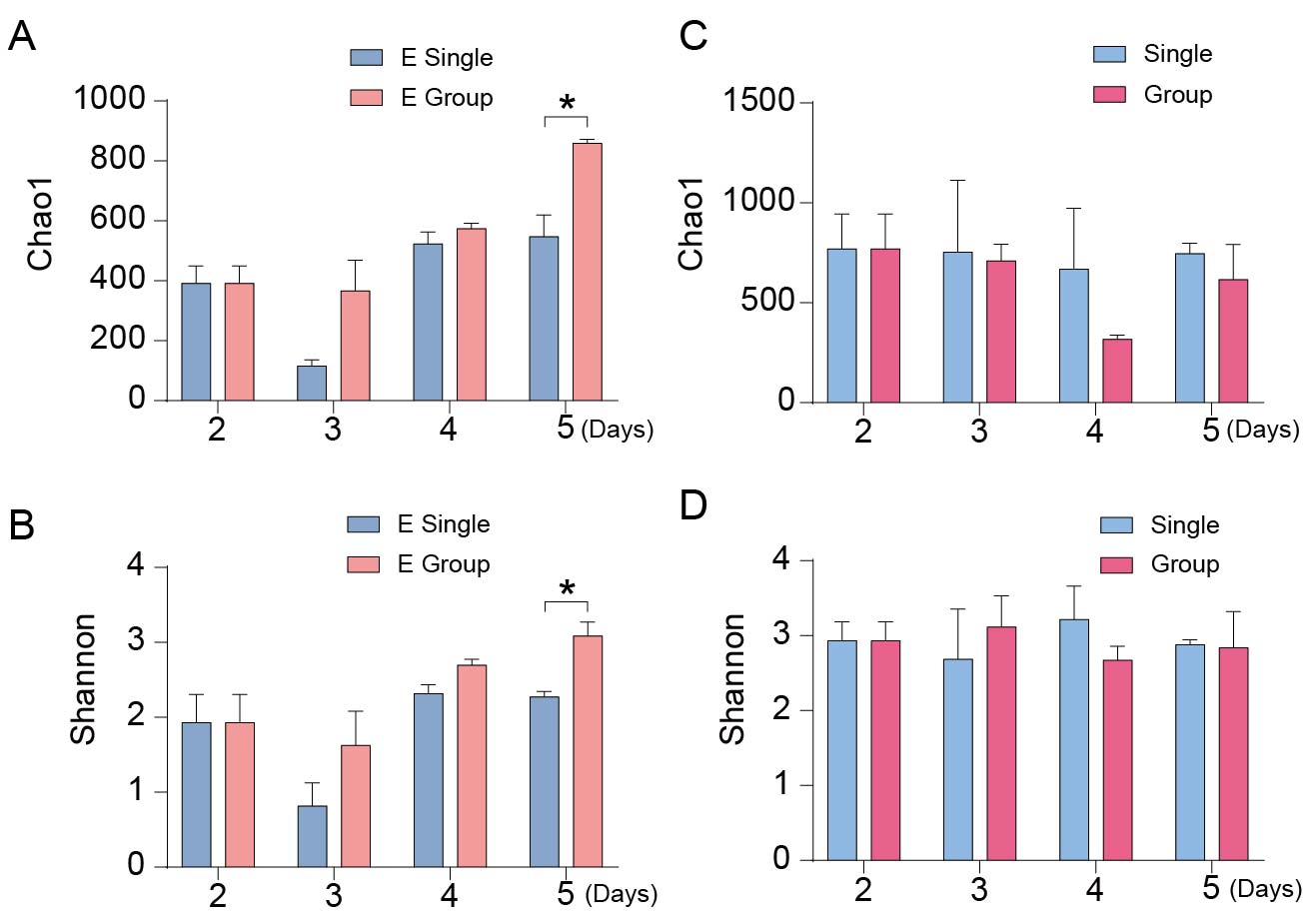


**Figure S2** **The alpha diversities of larval intestinal bacteria and environmental bacteria in feed under individual and group breeding condition**

**A-B.** The alpha diversities including Chao1 (**A**) and Shannon (**B**) index of environmental bacteria under single and group rearing on the 2^nd^, 3^rd^, 4^th^ and 5^th^ days. **C-D.** The alpha diversities including Chao1 (**C**) and Shannon (**D**) index of larval intestinal bacteria under single and group rearing on the 2^nd^, 3^rd^, 4^th^ and 5^th^ days.


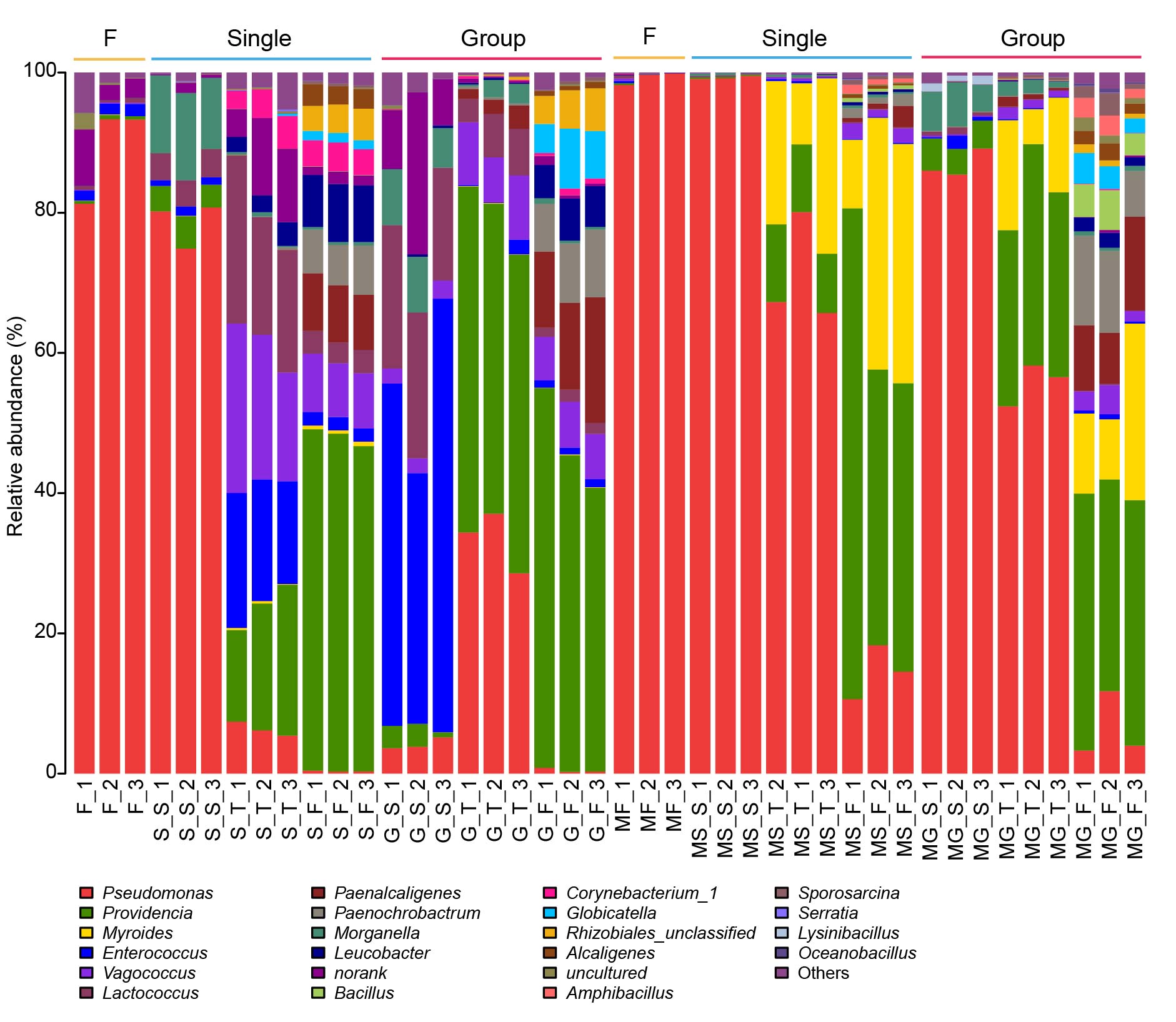


**Figure S3 The intestinal bacteria composition of house fly and environmental bacteria composition in feed under single and group breeding condition**

S: the larvae under single rearing; G: the larvae under group rearing; MS: the bran under single rearing condition; MG: the bran under group rearing condition. The “S”, “T” and “F” followed the S, G, MS and MG presented the second, third and fourth day of rearing. The number in the last of name presented the biological repeat. The relative abundances of all bacteria genera showed were over 1% in samples.


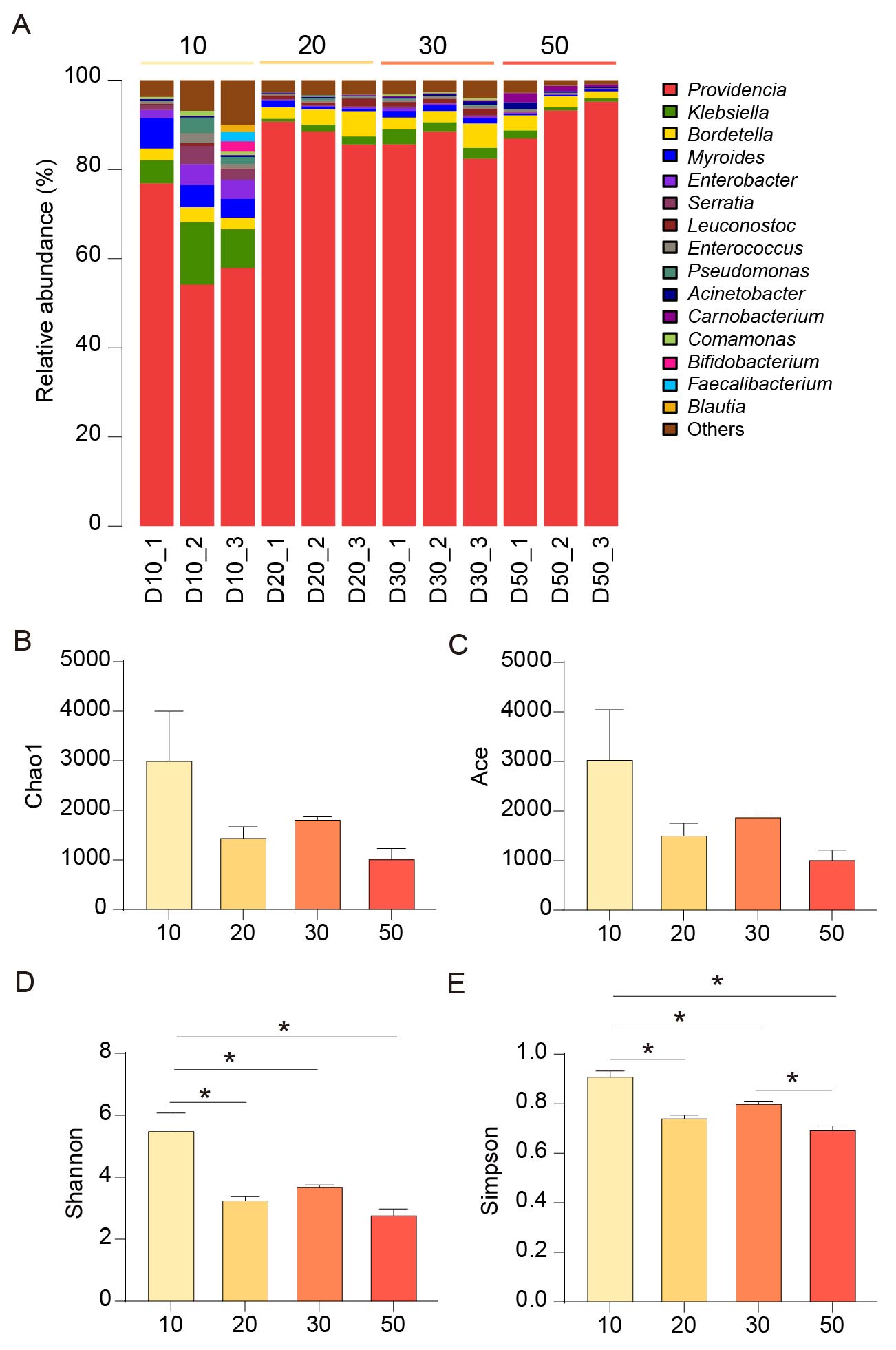


**Figure S4 Composition and diversity of gut microbiota in larvae under different population densities**

**A.** Composition of gut microbiota at the genus level in larvae at population densities of 10, 20, 30, and 50 individuals per tube. **B-E**. The alpha diversities including Chao1 (**B**), Ace (**C**), Shannon (**D**), and Simpson (**E**) index of intestinal bacteria of larvae at different population densities. Measurements in are shown as the mean ± SE. Statistical significance: *, P < 0.05.


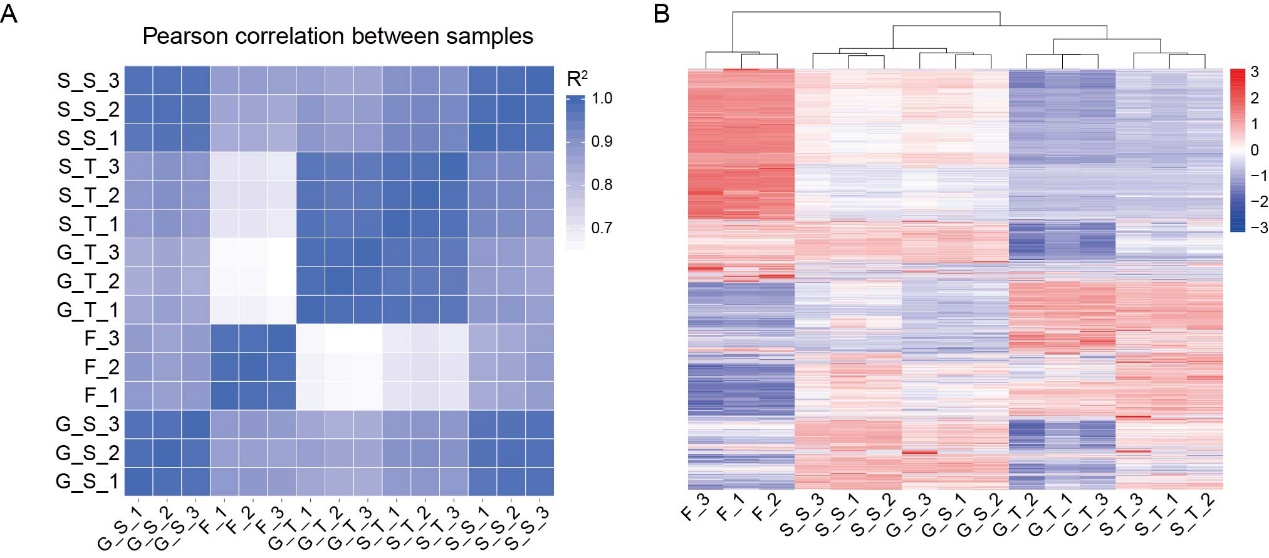


**Figure S5 Sample clustering and heatmap of differentially expressed genes**

**A.** The Pearson correlation of transcriptome samples. F: the larvae on the 1^st^ day of rearing; S_S/T: the larvae under single rearing on the 2^nd^/3^rd^ day; G_S/T: the larvae under group rearing on the 2^nd^/3^rd^ day. **B**. The expression heatmap of DEGs between single and group rearing on the 2^nd^ and 3^rd^ days.

Supplementary Tables:

**Table S1** Primers used in qPCR

| name | sequence | bacterium |
| --- | --- | --- |
| SerQf | TATTGCACAATGGGCGCAAG | *Serratia marcescens* |
| SerR1 | GGAGTTAGCCGGTGCTTCTT |  |
| gltA-F | CAGGCCGAATATGACGAATTC | *Klebsiella* |
| gltA-R | CGGGTGATCTGCTCATGAA |  |
| 16Shv-F | CTACGGGAGGCAGCAGTGG | *Pseudomonas* |
| 16Shv-R | TCGGTAACGTCAAAACAGCAAAGT |  |
| cloacae-F | CATGACACCGGTGTTTCCCCAGT | *Enterobacter* |
| cloacae-R | CGGTCGGTGAAGCCCAGAACCACTA |  |
| 341F | CCTAYGGGRBGCASCAG | Total bacteria |
| 806R | GGACTACNNGGGTATCTAAT |  |

**Table S2** T-test of data for three biological replicates of body weight

| Day | p value | t |
| --- | --- | --- |
| 2 | 0.00712580 | 5.071 |
| 3 | 0.59220905 | 0.5813 |
| 4 | 0.02725849 | 3.401 |

**Table S3** T-test of data for three biological replicates of body length

| Day | p value | t |
| --- | --- | --- |
| 2 | 0.00179539 | 7.382 |
| 3 | 0.30161647 | 1.185 |
| 4 | 0.03421079 | 3.159 |
